# The *fa2* gene and molecular markers mapping in the *gp* segment of the *Pisum* linkage group V

**DOI:** 10.1007/s13353-015-0335-0

**Published:** 2016-01-27

**Authors:** M. Gawłowska, W. Święcicki

**Affiliations:** Institute of Plant Genetics, Polish Academy of Sciences, Poznań, Poland

**Keywords:** *Pisum*, LGV, Stem fasciation, Chromosome mapping, Gene markers

## Abstract

Review studies on the world *Pisum* genetic resources have shown that stem fasciation is controlled by three loci, i.e., *fa1* (LGIV; Wt 10006 - type line of the Polish Gene Bank), *fa2* (LGV, the line Wt 12185), and *fas* (LGIII, the line Shtambovii). Outstanding advantages of this character (e.g., pods gathered in upper part of a stem) resulted in breeding some cultivars. Preliminary investigations suggested linkages of the newly described *fa2* gene within the *gp*–*U* segment. Based on the further linkage test crosses, it was stated that the *fa2* is localized between the *gp* and Pis_Gen_9_3_1 markers (in the LGV). Additionally, four molecular markers (AD175, AB146, AC58, and AD280) and the morphological marker *lk* were also localized in this segment. Moreover, *rms5*, *lum3*, and *cri* were found to map on the other side of *gp* with tight linkage observed between *lum3* and *cri*.

## Introduction

**Fig. 1:**
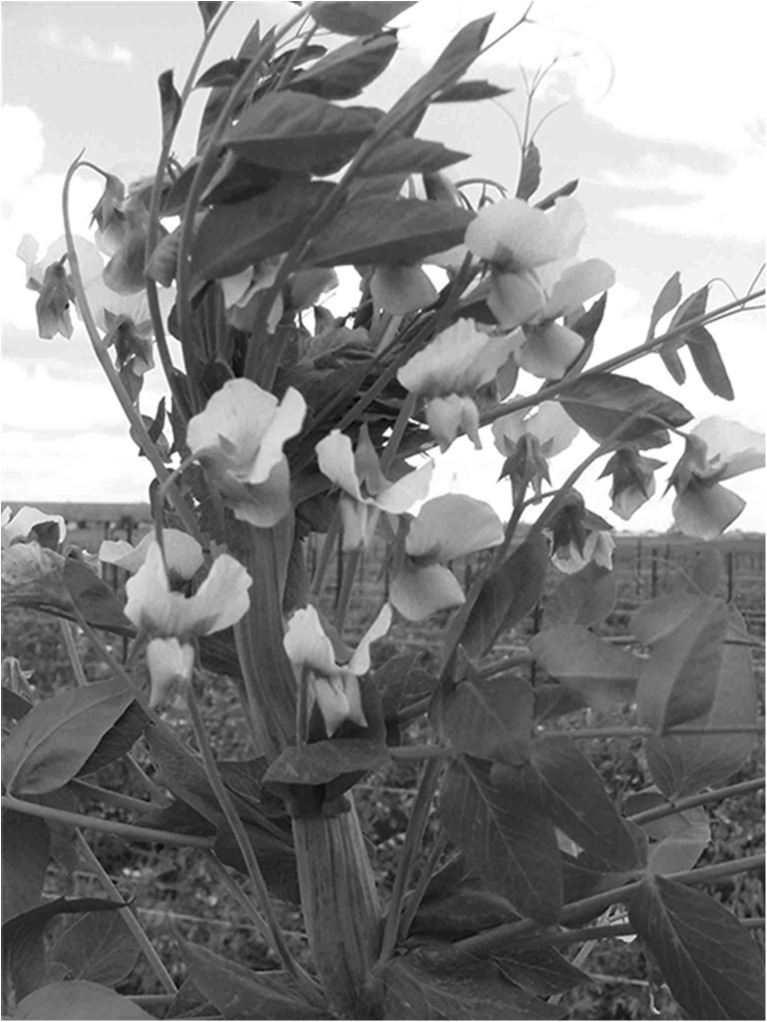
Stem fasciation in *Pisum* controlled by *fa1* or *fa2*

Stem fasciation in peas appears to be a very interesting character from a theoretical as well as a practical point of view. This character not only changes the stem architecture but also the physiology of flowering and maturing (Fig. 1). Its advantage is that pods are gathered in upper part of a stem; but in consequence, pea plants lodge and are susceptible to drought during shortened flowering and pod formation periods. Outstanding advantages resulted in breeding some cultivars, for example, cvs. Buława (POL), Ornamenta, Rosacrone, Golf (DEU) and Novella (USA). Pea fasciation was described for the first time in 1597 (Święcicki 2001, after Derbshire 1911), and since then different names have been used for its designation, such as the *Pisum umbellatum*, mummy pea, or crown pea. Furthermore, a taxon was separated in *Pisum* taxonomy, i.e., *P. sativum,* subsp. *sativum*, convar. vulgare var. *coronatum* (Lehmann and Blixt 1984).

**Fig. 2:**
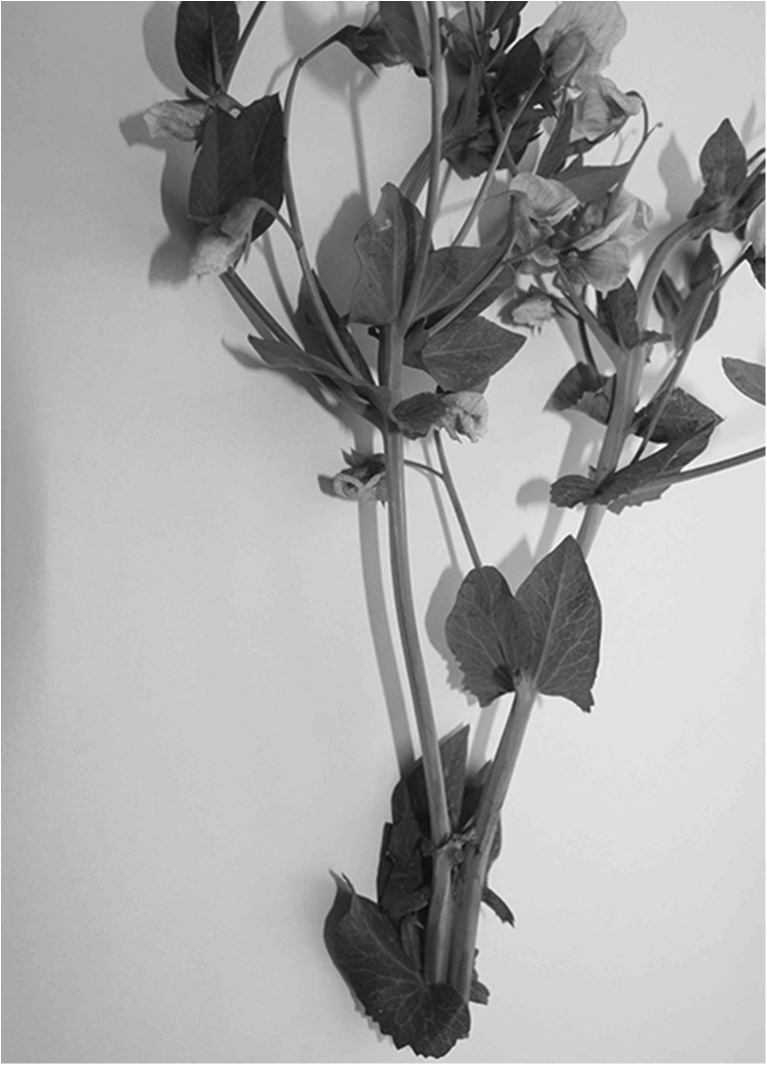
Stem *dichotomous branching* in *Pisum* controlled by *fa1* ^*bif*^ gene

Fasciation was one of the seven monohybrid characters studied by Mendel (Święcicki et al. 2000). Induced mutations resulted in a number of independent mutation cases with a similar phenotype in different genotypic backgrounds which are still maintained in world *Pisum* collections (e.g., USDA Pullman Pisum Genetic Stock Collection, John Innes Centre Pisum Collection, Wiatrowo Pisum Collection). Information on the character anatomy, morphology, and expression are available in several references such as Gottschalk and Wolf (1983), Marx and Hagedorn (1962) and Sinjushin and Gostimsky (2006), but different opinions are available on its mode of inheritance. It has been shown that this character is controlled by one to four independent genes or multiple alleles of a single locus (Marx and Hagedorn 1962; Blixt 1972; Lamprecht 1974; Święcicki 2001). The most popular was the acceptance of two independent fasciata genes — *fa* in LG IV and *fas* in LG III (Lamprecht 1974; Blixt 1977). Additionally, a similar mutation type, *dichotomous branching*, was selected and reported as a character governed by two polymeric genes *bif1* and *bif2* (Gottschalk and Wolf 1983); this alteration was associated with a fasciation of only a few upper nodes that results in a forked stem (Fig. 2). Results of subsequent complementation tests (locus identity test crosses) that explain the genetic basis of fasciata phenotype in pea lines from the Blixt’s, Gottschalk’s, Marx’s, Święcicki’s and Sinjushin’s collections were as follows (Święcicki 2001; Sinjushin et al. 2006):

- no typeline registered by Blixt (1977) exists in the main *Pisum* collections for the *fas* gene from LGIII; Sinjushin et al. (2006) and Sinjushin (2011) suggested that the two lines, JI2771 and mutant *Shtambovii*, have the *fas* gene,
- *fasciation* in most of the tested lines is controlled by the *fa* gene from LGIV,
- *dichotomous branching* appeared to be controlled by the allele in the *fa* locus (symbol *fa*^*bif*^ was suggested),
- an exception is the fasciation in the accession Wt 12185 as controlled by a gene different from the *fa* locus; for *fa* in LGIV, the symbol *fa1* (and *fa1*^*bif*^) was suggested and *fa2* for the new gene in the type line Wt 12185.

For usage of a stem fasciation in breeding, the results of Gottschalk (1979) and Święcicki (2001) are important, indicating that lines Gott37B (*fa1*^*bif*^) and Wt12185 (*fa2*) are characterised by a full penetrance of mentioned genes and an increased seed production.

Preliminary results have shown that the new *fasciata* gene, *fa2,* is linked with *gp* in linkage group V (LGV) (Cr0 = 17.6 ± 7.6) (Święcicki and Gawłowska 2004). The aim of this study was to map *fa2* using more markers from the *gp* region, including molecular markers.

## Material and methods

For the purpose, the type line for the gene *fa2* (Wt 12185) from the Polish *Pisum* Gene Bank at Wiatrowo was selected. For the linkage test crosses, this line was crossed to tester lines with LGV morphological markers, particularly from the *gp* region: Wt 10498: *lum3*, *cri* (cross number: K. 3365); Wt 15294: *gp, rms5* (K. 3548 and K. 3048); Wt 15860: *creep*, *ce* (K. 3528); Wt 10287: *lk* (K. 3364); and Wt 11238: *gp, cp1* (K. 3338). Phenotypic expression of the markers is shortly given below:

- *Gp*–*gp* = green–yellow color of pods (*luteo-legumina* mutation type, Blixt 1977)
- *Rms5*–*rms5* = plant with one stem- with many basal branches (*ramosus*, Apisitwanich et al. 1992)
- *Creep*–*creep* = perpendicular–creeping stem growth (*creeping*, Blixt 1977)
- *Ce*–*ce* = anthocyanin/cardinal–cherry flower color (*cerise*, Blixt 1977)
- *Lk*–*lk* = normal–dwarf plant plus other complex changes (*erectoides*, Święcicki 1989)
- *Lum3*–*lum3* = interveinal leaf areas green–yellowish (*costata,* Świecicki 1988)
- *Cri*–*cri* = leaf and stipules surface smooth–crinkled (*crispa*, Blixt 1977)
- *Cp1*–*cp1* = pods straight–concavely curved (*concavum*, Blixt 1977)

Additionally, a segregation of molecular markers was analysed: Pis_Gen_9_3_1 in K. 3548, Pis_Gen_9_3_1, AD175, AB146, AC58 in K. 3319, and Pis_Gen_9_3_1, AD280, AC58 in K. 3338.

The Pis_Gen_9_3_1 marker (the abbreviation Pis_Gen_9_3 is used in the text) is a sequence tagged site marker obtained in a framework of the EU Grain Legumes Integrated Project (2004–2008). The primer information is available on the following website: http://bioweb.abc.hu/cgi-mt/pisprim/pisprim.pl. A polymorphism was identified after sequencing [A/G, SNP 312 *bp*] and visualized after *AsuI* digestion. PCR was conducted as follows: 1× buffer Go-Taq flexi (Promega, Madison, WI, USA), 1.5 mM MgCl_2_ (Promega), 1 mM dNTP (ThermoFisher Scientific, Waltham, MA, USA), primer 1 (1 μM), primer 2 (1 μM), 0.6 U GoTaq polymerase (Promega), 100 ng/μl BSA (Sigma-Aldrich, St. Louis, MO, USA), and 25 ng DNA. PCR temperature profile TD 60–40 °C was used (Hecker and Roux 1996).

SSR primers were designed by the Pea Microsatellite Consortium, Agrogene, France, and used in mapping by Loridon et al. (2005). Five molecular markers were used showing a polymorphism in investigated populations and linkages with the investigated LGV (Pis_Gen_9_3; Święcicki et al. 2012 and AB146, AC58, AD175, AD280; Loridon et al. 2005). PCR was conducted as follows: 1× buffer GoTaq flexi (Promega), 1.5 mM MgCl_2_ (Promega), 0.6 mM dNTP (ThermoFisher Scientific), primer 1 (0.2 μM), primer 2 (0.2 μM), 0.6 U GoTaq polymerase (Promega), and 25 ng DNA. PCR profile: 95 °C (2 min); 35× [95 °C (30 s)], required TM [AB146 (64 °C), AD175 (58 °C), AD280 (64 °C), AC58 (58 °C) (60 s)], 72 °C (60 s), and 72 °C (5 min).

The results of mono- and dihybrid segregations were calculated using a computer program based on the product-ratio method for linkage estimation (Święcicki et al. 1998). For graphic presentation of the loci order, the MapChart program was used (Voorrips 2002).

## Results and discussion

Mono- and dihybrid segregations in the F_2_ generation of seven populations were analysed by the *fa2* gene and eight morphological and five molecular markers (Table 1). Preliminary results suggest that most of the selected markers originate from the *Gp*–*Fa2*–*U* region (Święcicki and Gawłowska 2004). Markers *lum3, cri*, and *cp-1* localized on the opposite side of *gp* (Blixt 1977; Święcicki 1988; Weeden et al. 1998) additionally should confirm a selection of appropriate chromosome region for the *fa2* localization. And it appeared (Table 2, K. 3365) that for gene pairs *Fa2*–*Cri* and *Fa2*–*Lum3,* there were no deviations from correct dihybrid segregation and no linkages. A valuable result, hitherto not known, is the strong linkage revealed for *Lum3*–*Cri* (Cr-0 = 1.9).

**Table 1:** Monohybrid segregation for the investigated gene *fa*2 and gene markers in LGV observed in the *F2* population of the linkage test crosses (see footnote)

Cross combination	Gene	Allele		Total chi square (3:1)	
		Dominant	Recessive		
K. 3365	*Lum3*	86	23	109	0.88
	*Cri*	86	23	109	0.88
K. 3548	*Fa2*	152	42	194	1.16
K. 3048		261	65	326	4.45
K. 3528		194	49	243	3.00
K. 3364		204	65	269	0.10
K. 3365		84	22	106	1.02
K. 3548	*Gp*	148	47	195	0.08
K. 3048		243	82	325	0.01
K. 3319		80	24	104	0.20
K. 3338		102	40	142	0.76
K. 3548	*Rms5*	148	47	195	0.08
K. 3048		263	86	349	0.02
K. 3528	*Creep*	199	54	253	1.80
	*Ce*	134	47	181	0.90
K. 3364	*Lk*	223	63	286	1.35
K. 3338	*Cp-1*	85	32	117	0.34
K. 3548	Pis_Gen_9_3	49	19	68	0.31
K. 3319		69	22	91	0.03
K. 3338		86	36	122	1.32
K. 3319	AD 175	63	23	86	0.14
	AC 58	63	17	80	0.60
K. 3338		94	22	116	2.25
K. 3319	AB 146	59	11	70	3.22
K. 3338	AD 280	73	24	97	0.00

Linkage test crosses: K. 3548 = Wt 12185 × Wt 15294, K. 3048 = Wt 15294 × Wt 12 185, K. 3528 = Wt 12185 × Wt15860, K. 3364 = Wt 12 185 × Wt 10287, K. 3319 = Wt 3527 × Wt 11238, K. 3365 = Wt 12185 × Wt 10498, K. 3338 = Wt 15989 × Wt 11238

**Table 2:** Distrubution phenotypes in F_2_ populations and the linkage test for the *fa2* gene (Wt 12185—typeline × tester lines)

Cross combination	Pair of genes	Phase	Phenotype				Total	Joint chi squere	Cr-0 value ± SE (per cent)
			DD	Dr	rD	rr			
K.3365	*Fa2-Lum3*	R	60	20	19	3	106	1.3	40.4 ± 8.0
	*Fa2-Cri*	R	60	20	19	3	106	1.3	40.4 ± 8.0
	*Lum3-Cri*	C	86	0	0	23	109	105.6	1.9 ± 1.3
K. 3548	*Fa2-Gp*	R	105	47	42	0	194	16.7	15.8 ± 6.9
	*Fa2-Rms5*	R	106	46	42	0	194	16.8	16.0 ± 7.0
	*Fa2-* Pis_Gen_9_3	C	48	9	1	10	68	26.7	10.8 ± 4.0
	*Rms5-Gp*	C	144	4	4	43	195	159.1	4.2 ± 1.5
	*Rms5-*Pis_Gen_9_3	R	28	16	21	3	68	5.0	31.2 ± 10.8
	*Gp*-Pis_Gen_9_3	R	26	16	23	3	68	5.0	29.2 ± 10.9
K. 3048	*Fa2-Gp*	R	179	81	64	1	325	23.2	12.9 ± 5.4
	*Fa2- Rms5*	R	183	78	63	2	326	20.3	18.5 ± 5.3
	*Rms5-Gp*	C	234	11	9	71	325	229.6	6.3 ± 1.4
K. 3528	*Creep- Fa2*	R	141	49	53	0	243	17.9	15.9 ± 6.2
	*Creep- Ce*	C	117	27	17	20	181	17.3	29.1 ± 4.1
	*Fa2-Ce*	R	96	43	37	4	180	7.9	30.7 ± 6.6
K. 3364	*Fa2- Lk*	R	147	57	65	0	269	23.6	13.8 ± 6.0
K. 3319	*Gp-*Pis_9_3	C	62	6	5	16	89	41.2	13.4 ± 3.9
	*Gp-*AD175	C	59	4	2	19	84	53.0	6.9 ± 2.9
	*Gp-*AC58	C	55	4	6	13	78	33.6	14.0 ± 4.3
	*Gp-*AB146	C	55	3	2	8	68	27.2	9.3 ± 3.7
	Pis_Gen_9_3-AD175	C	59	3	2	19	83	53.7	6.0 ± 2.7
	Pis_Gen_9_3- AC58	C	54	4	7	12	77	27.0	15.7 ± 4.6
	Pis_Gen_9_3-AB146	C	56	0	1	10	67	48.4	3.6 ± 2.3
	AD175-AC58	C	54	2	7	14	77	41.5	10.7 ± 3.8
	AD175-AB146	C	58	1	0	10	69	48.4	3.5 ± 2.2
	AC58-AB146	C	50	3	2	6	61	32.2	11.1 ± 4.3
K. 3338	*Cp1*-*Gp*	C	74	10	8	24	116	47.6	15.9 ± 3.8
	*Cp1*- Pis_9_3	C	63	10	10	21	104	31.9	19.9 ± 4.5
	*Cp1*-AD280	R	44	16	24	2	86	3.1	30.1 ± 9.7
	*Cp1*-AC58	C	56	13	22	6	97	0.3	47.7 ± 7.4
	*Gp*- Pis_Gen_9_3	C	81	6	5	30	122	77.9	8.9 ± 2.7
	*Gp-* AD280	R	47	21	26	3	97	4.3	31.6 ± 9.0
	*Gp-* AC58	C	70	15	24	7	116	0.3	45.7 ± 6.6
	Pis_Gen_9_3-AD280	R	44	22	27	2	95	6.7	25.1 ± 9.5
	Pis_Gen_9_3-AC58	C	71	9	21	12	113	10.3	30.4 ± 5.4
	AC58-AD280	R	52	20	15	1	98	3.7	26.8 ± 9.8

Joint segregation of gene pairs in chromosome 5 (K. 3365 = Wt 12185 × Wt 10498), K. 3548 = 12185 × Wt 15294, K. 3048 = Wt 15294 × Wt 12185, K. 3528 = Wt 12185 × Wt 15860, K. 3364 = Wt 12185 × Wt 10287, K. 3319 = Wt 3527 × Wt 11238, K. 3338 = Wt 15989 × Wt 11238

**Fig. 3:**
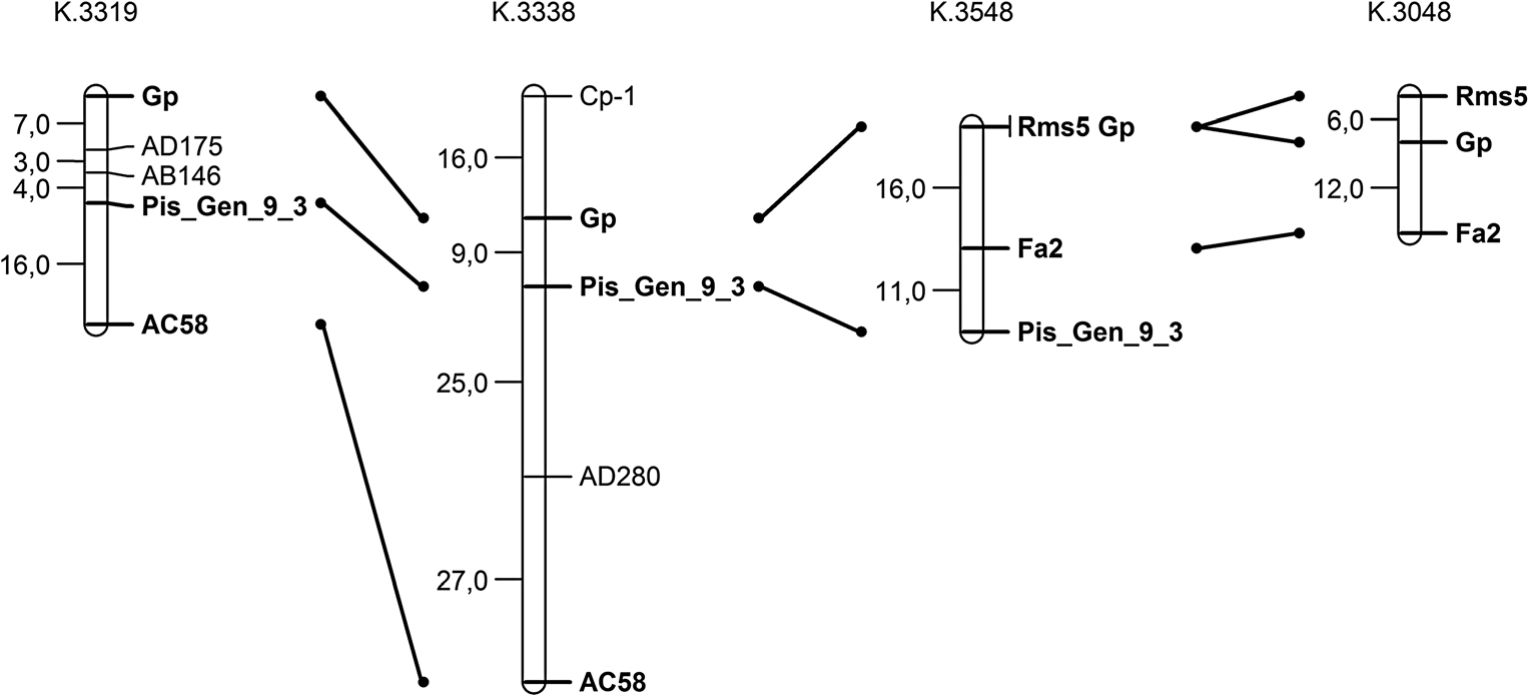
Loci order including *fa*2 in the *gp* segment of the LGV in four mapping populations

Correct, monohybrid segregation for the *fa2* and selected markers (Table 1) allowed us to analyse a dihybrid segregation to look for linkages and the *fa2* locus (Table 2). In K. 3548 and K. 3048 populations (reciprocal crossings), *fa2* and markers *gp, rms5,* Pis_Gen_9_3 and *gp, rms5*, segregated respectively. Substantial deviations from a dihybrid segregation for most of the gene pairs were stated. Exceptions were as follows: *rms5—*Pis_Gen_9_3 and *gp—*Pis_Gen_9_3 with Cr-0 values of about 30. Taking into account Cr-0 values obtained from the K. 3548 population, the following loci order can be accepted: *Gp*/*Rms5*–*Fa2*– Pis_Gen_9_3. The supplemental analysis of the K. 3048 population allows us to accept the gene order presented in Fig. 3. Substantial deviations from correct dihybrid segregation and linkages for *Fa2*–*Creep* and *Creep*–*Ce* and no deviations for *Fa2*–*Ce* (K. 3528 population, Table 2) additionally confirm the *fa2* localization and the presented loci order (see also consensus *Pisum* map, Weeden et al. 1998). A valuable supplement for this region is the linkage *Fa2*–*Lk* = 13.8, revealed in the population K. 3364. Together with the earlier result for *Gp*–*Lk* = 12.3 (Święcicki 1989), it is emphasized that the *Lk* gene is also localized in the investigated *Gp*–*U* region.

Supplemental linkage data in the above-mentioned region supply an analysis of K. 3319 and K. 3338 populations covering the *Gp* locus and five molecular markers: Pis_Gen_9_3, AD175, AB146, AC58, and AD280. Obtained results suggest the loci order given in Fig. 3.

Conducted analyses localized the new *fa2* gene in the *Gp*–*U* segment of the LGV between *Gp* and Pis_Gen_9_3 markers. Four additional molecular markers (AD175, AB146, AC58, AD28) and morphological *lk* were also localized in this segment. Moreover, the locus *rms5* and a strong linkage between *lum3* and *cri* were found from the other side of the *Gp* locus.
